# Age-dependent motor unit remodelling in human limb muscles

**DOI:** 10.1007/s10522-015-9627-3

**Published:** 2015-12-14

**Authors:** Mathew Piasecki, Alex Ireland, David A. Jones, Jamie S. McPhee

**Affiliations:** School of Healthcare Science, Manchester Metropolitan University, Manchester, M15GD UK

**Keywords:** Motor neuron, Motor unit, Ageing, Muscle, Sarcopenia

## Abstract

Voluntary control of skeletal muscle enables humans to interact with and manipulate the environment. Lower muscle mass, weakness and poor coordination are common complaints in older age and reduce physical capabilities. Attention has focused on ways of maintaining muscle size and strength by exercise, diet or hormone replacement. Without appropriate neural innervation, however, muscle cannot function. Emerging evidence points to a neural basis of muscle loss. Motor unit number estimates indicate that by age around 71 years, healthy older people have around 40 % fewer motor units. The surviving low- and moderate-threshold motor units recruited for moderate intensity contractions are enlarged by around 50 % and show increased fibre density, presumably due to collateral reinnervation of denervated fibres. Motor unit potentials show increased complexity and the stability of neuromuscular junction transmissions is decreased. The available evidence is limited by a lack of longitudinal studies, relatively small sample sizes, a tendency to examine the small peripheral muscles and relatively few investigations into the consequences of motor unit remodelling for muscle size and control of movements in older age. Loss of motor neurons and remodelling of surviving motor units constitutes the major change in ageing muscles and probably contributes to muscle loss and functional impairments. The deterioration and remodelling of motor units likely imposes constraints on the way in which the central nervous system controls movements.

## Introduction

The motor unit (MU) consists of a single alpha motor neuron and all of the muscle fibres it innervates (Sherrington 1925) and is the smallest functional component of the neuromuscular system. The motor neuron cell body is located in the ventral horn of the spinal cord and extends an axon that branches to form a neuromuscular junction at the site of innervation with individual muscle fibres. The muscle fibres within a MU all have the same phenotypic characteristics (i.e., slow or fast, type I or type II) and are activated together in an all-or-none manner. MU territories are distributed over several cm length (Gallina and Vieira 2015; Héroux et al. 2015) and around 5–10 mm depth in large limb muscles (Buchthal et al. 1959) to innervate fibres in a mosaic pattern. Around two-dozen individual MUs with innervation ratios of around 500 to 2000 fibres can span a muscle cross-sectional depth of around 8 mm (Buchthal et al. 1959). Thus, fibres of the same MU are rarely ever located immediately adjacent to one another in order to ensure distribution of forces across relatively large areas of muscle and repetitive extracellular depolarisations are less likely to be intense in any one area upon activation (Edström and Larsson 1987).

The first dorsal interosseous (FDI), a small hand muscle, is estimated to have around 120 MUs and 40,000 muscle fibres (Feinstein et al. 1955), while larger limb muscles can each contain many hundreds or thousands of MUs of varying sizes innervating over a million muscle fibres per muscle (e.g. see Tomlinson and Irving 1977; Lexell et al. 1988). The precise innervation ratios of MUs are difficult to estimate and vary considerably between MUs within a single muscle (Enoka and Fuglevand 2001), the variations being directly proportional to the force generated as demonstrated in rat muscles (Kanda and Hashizume 1992; Tötösy de Zepetnek et al. 1992). Estimations of innervation ratios in the biceps brachii (BB) indicate 209–750 (Buchthal et al. 1959; Gath and Stålberg 1981), the tibialis anterior averages around 329–562 (Feinstein et al. 1955; Gath and Stålberg 1981) and deltoid averages 339 fibres per MU (Gath and Stålberg 1981). By estimating number of motor neurons and number of muscle fibres, Feinstein (Feinstein et al. 1955) estimated average innervation ratios at around 340, 410 and 1934 in FDI, brachialis and medial gastrocnemius, respectively.

In order to know how the MU changes with ageing, it is necessary to have methodologies to assess their numbers and functions. There are no techniques currently available to directly count MUs in healthy humans, so efforts have been restricted to post-mortem anatomical estimates or electromyography (EMG). EMG enables detailed investigations of MU function and recruitment patterns as well as estimates of their numbers in individual muscles. The range of techniques available to estimate MU numbers using EMG in humans have been reviewed elsewhere (Daube 2006; Bromberg 2007; Gooch et al. 2014), hence they will be described only briefly here. Instead, the aim of this review is to summarise current evidence to indicate the extent of MU remodelling during healthy human ageing and the possible consequences for control of movements.

## Methods to estimate motor unit numbers in humans

### Anatomical counts of motor neurons and muscle fibres

Post mortem anatomical studies indicate neurological changes with ageing. Tomlinson and Irving (1977) examined the lumbosacral spinal cord of 47 deceased specimens aged 13–95 years. Estimates of motor neuron cell bodies remained relatively constant until the age of around 60, after which they declined progressively such that specimens from those aged around 75 had approximately 30 % fewer motor neurons to their lower limbs (Kawamura et al. 1977; Tomlinson and Irving 1977; Mittal and Logmani 1987). These anatomical studies leave no doubt that neurons in the spinal cord are lost with age, but it is difficult to distinguish between motor and sensory neurons in anatomical counts.

### Electromyography

When a muscle fibre receives an impulse from a nerve the permeability of the fibre membrane to sodium is temporarily increased, which reverses the membrane potential of the muscle fibre. The action potential propagates along fibres and can be detected with electrodes and an appropriate amplifier.

McComas et al. (1971) first reported EMG-based techniques for motor unit number estimates (MUNE) in volunteers aged 4–58 years. The nerve branch innervating the extensor digitorum brevis (EDB) in the foot was stimulated percutaneously and the surface-recorded compound muscle action potentials (CMAP) were captured over the muscle belly. The average MU potential was calculated from all of the measured MU potentials and this value was divided into a maximal CMAP, recorded after supramaximal stimulation of the motor neuron branch, in order to derive a MUNE value. A problem with this technique is *alternation*, which occurs due to inconsistent activation of different MUs with similar activation thresholds, and cross-contamination from nearby muscles that share the main nerve pathway (McComas et al. 1993). Another limitation of all techniques that require a maximal CMAP is that they can only be performed on superficial muscles that have a major nerve branch accessible superficially to be stimulated percutaneously.

**Fig. 1:**
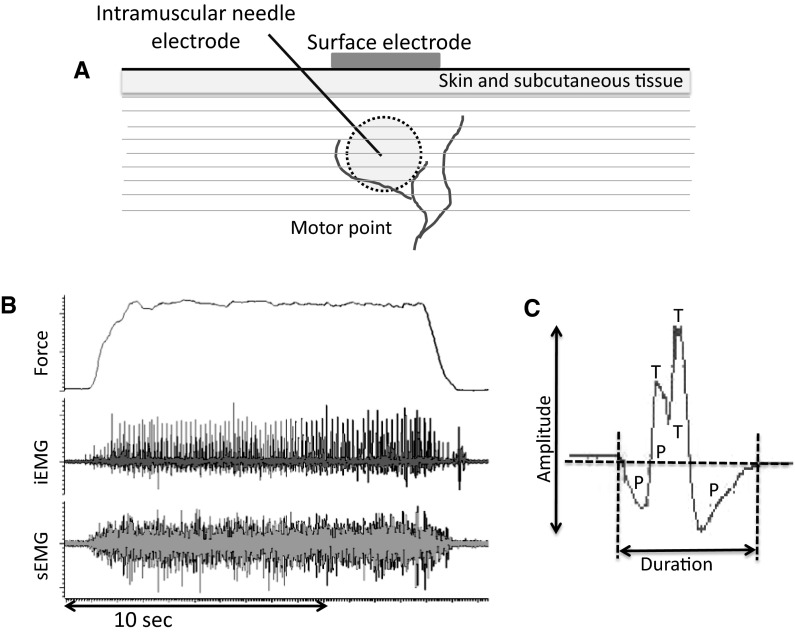
Motor unit recordings. **a** Schematic showing surface and indwelling needle electrodes at the muscle motor point. A typical concentric needle electrode may capture MUPs from up to around 2000 muscle fibres. **b** Raw data recorded from the vastus lateralis of a healthy older man showing force, intramuscular and surface EMG traces. **c** A single motor unit potential showing amplitude, duration and complexity in terms of phases (*P*) and turns (*T*)

More recent techniques overcame the problem of alternation by recording motor unit potentials (MUPs) during low and moderate intensity voluntary contractions (Brown et al. 1988). An indwelling needle electrode (iEMG) records from a small proportion of muscle fibres within individual MUs and the MUPs are used to ‘trigger’ the corresponding surface-recorded MU potential to generate a surface MUP (sMUP): a technique known as ‘spike triggered averaging’ (STA). An average sMUP is generated from around 20 sMUPs; this is then divided into the maximal CMAP to derive a MUNE value. The sEMG needs to be recorded over the motor point where the cluster of motor axons gives fast and reliable rise-times for the CMAP and sMUPs, and the MUPs and sMUPs are more likely to be time-locked as they are ‘seen’ by both electrodes at the same time (Fig. 1) (Brown et al. 1988). The use of an indwelling electrode (needle or fine-wire) makes the STA technique invasive, but this is only a minor inconvenience for most adults because the needles used (around 26 gauge) are often smaller than those used to collect routine blood samples.

The complex iEMG and sEMG signals from voluntary contractions can be separated into the constituent MUPs from individual MUs using automated signal decomposition software, which removes most of the subjective and laborious interpretations (Stashuk 1999a; Abdelmaseeh et al. 2014; Gooch et al. 2014). However, the EMG signals are increasingly complex with higher intensity contractions, causing difficulties when decomposing into MUs during collections from high intensity contractions. Thus, most studies report STA-MUNE values during moderate intensity contractions. According to the Henneman size principle (Henneman et al. 1965) and evident also in human muscles (Milner-Brown et al. 1973), MUs recruited during low-force contractions are the smallest and produce the lowest forces. STA-MUNE will therefore disproportionately sample from the early-recruited, smaller MUs and subsequently give an excessively high MUNE value.

Another technique was developed recently, known as the motor unit number index (MUNIX) (Nandedkar et al. 2010). It does not use intramuscular measurements, relying only on the surface-recorded interference pattern to reach a MUNIX value. The ‘power’ and ‘area’ of the sEMG signal from a maximal CMAP is compared with those from the surface interference pattern (SIP) obtained during voluntary contractions at different intensities. Using this technique, sarcopenic patients had ~30 % lower values in the hypothenar than non-sarcopenic adults (Drey et al. 2013; Drey et al. 2014). This method has so far mainly focussed on the small peripheral muscles like the thenar (Nandedkar et al. 2010; Li et al. 2012; Zhou et al. 2012; Furtula et al. 2013). Correlations with other MUNE methods have been reported (Furtula et al. 2013), although MUNIX still needs to be systematically validated against other more established MUNE measures. The major limitations with MUNIX, however, are that the extent of signal attenuation (discussed below) might differ between young and old and therefore affect any between-group comparisons, and the lack of iEMG data means that individual MUs cannot be characterised.

## Ageing-related changes to motor unit numbers

The first study to demonstrate MU loss during healthy ageing using EMG was by Campbell et al. (1973). They used the incremental stimulation technique to study the EDB in 94 subjects aged 3–96 years. In agreement with the anatomical counts by Tomlinson and Irving (1977), MUNE remained relatively constant (mean = 197 ± 58) up to the age of around 60 years, but a progressive decline was noted thereafter. People aged over 75 years had fewer than 50 % of the MUs compared with young and some of the very oldest subjects apparently had fewer than 10 % of their MUs remaining. Similarly, the multi-point stimulation technique showed around 50 % fewer MUs and smaller CMAP in the thenar muscles of 20 older adults (63–81 years) compared with 17 young (21–28 years) (Doherty and Brown 1993). These EMG techniques indicate much greater loss of MUs than was reported from anatomical counts by Tomlinson and Irving (1977).

**Fig. 2:**
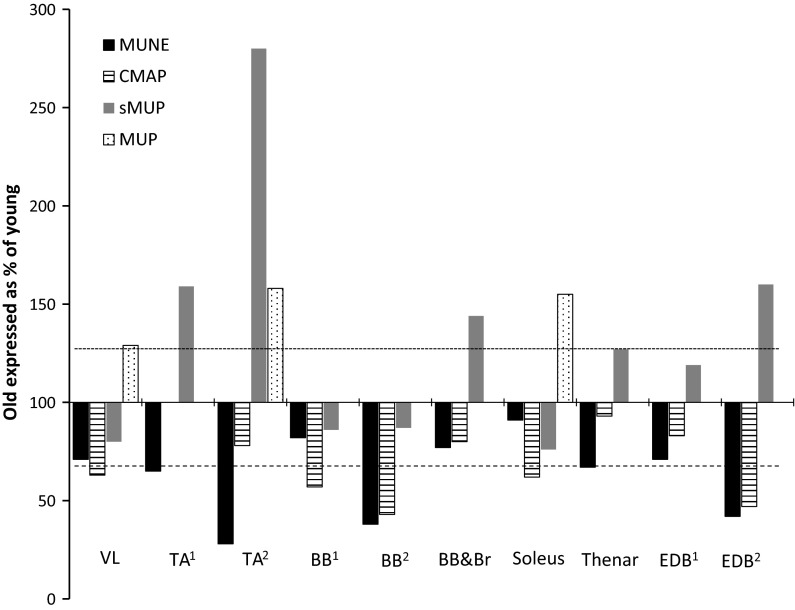
MUNE values, CMAP, sMUP and MUP values in old compared with young. The *dashed horizontal line* indicates the median MUNE in old and the dotted *horizontal line* indicates the median MUP or sMUP in old expressed as % of young from the various studies. Where multiple older age groups or contraction intensities were reported in a single study, the age range 60–80 years and closest intensity to 25 % MVC were used in this figure. *TA* Tibialis anterior, *BB* biceps brachii, *BB&Br* biceps brachii and brachialis, *EDB* extensor digitorum brevis. Data are from: VL (Piasecki et al. 2015) TA^1^ (Power et al. 2010); TA^2^ (Hourigan et al. 2015); TA^3^ (Dalton et al. 2008); BB^1^ (Galea 1996); BB^2^ (Power et al. 2012); BB&Br (Brown et al. 1988); Soleus (Dalton et al. 2008); Thenar (Galea 1996); EDB^1^ (Galea 1996) and EDB^2^ (Campbell et al. 1973)

More recent studies using the latest STA-MUNE techniques with decomposition-enhanced quantitative signal processing showed MUNE values to be lower in older people, although the vast majority of work focussed on small, peripheral muscles controlling the hand or foot. The results of selected studies that used incremental stimulation or STA-MUNE are summarised in Fig. 2. Across the various studies that examined different muscles the median MUNE value of old was 66 % (dashed horizontal line in Fig. 2) and the sMUP or MUP was 149 % (dotted horizontal line in Fig. 2) of the value of young.

## Cross-contamination, attenuation and recording area of the sMUPs and CMAP

MUNE values are a widely used representation of MU numbers, but the reliance upon sEMG for sMUPs and the CMAP has several limitations, including cross-contamination and attenuation of electrical signals and the problems of recording from representative groups of MUPs and sMUPs.

### Cross-contamination in small muscles

A supramaximal percutaneous stimulation to the motor nerve assumes that all MUs in the muscle will be activated and the ensuing maximal CMAP is a function of the total number of MUs within the muscle (Kurokawa et al. 1999; Wee 2006; Severinsen and Andersen 2007). When sampling from small muscles, the CMAP values can suffer from cross-contamination of electrical activity from other nearby muscles. One study estimated that in the small abductor digiti minimi, more than 60 % of the sMUPs originated from other nearby hand muscles (Kawamura et al. 2013), thereby complicating the interpretation of the sMUPs and MUNE values.

### Attenuation of electrical signals in large muscles

A consistent finding is that the CMAPs recorded from older subjects are smaller than those from young (Fig. 2). This is unlikely to be due to fibre atrophy because disuse in young subjects did not change CMAP amplitude in the soleus (Clark et al. 2006) or the FDI (Fuglevand et al. 1995). If the CMAP is the summation of all electrical activity within the recording area, the lower CMAP in the old may be the result of relatively fewer muscle fibres. But, there are other considerations.

Older muscle can have higher intramuscular fat and connective tissue (Lexell et al. 1988; Hogrel et al. 2015), possibly causing diminution of signals recorded at the surface. Studies of MUNE in older age have not systematically considered signal interference, but the effects are relatively easily estimated by examining MUPs and their corresponding sMUPs. Assuming the MUP size is proportional to the total cross sectional area (CSA) of muscle fibres within a MU (Rosenfalck 1969), and therefore indicative of MU size, two similar MUPs recorded from a similar depth should have similar sMUPs if no attenuation of the signal occurred. Data collected from a small group of young men (unpublished) can be used to illustrate the point. A series of MUPs averaging 1159 µV ms recorded from a superficial region of the vastus lateralis (VL) gave a sMUP of 525 µV ms. Slightly larger MUPs of 1280 µV ms recorded from the deepest part of the same muscle (around 2 cm deep) gave an average sMUP that was 513 µV ms. Overall, the superficial MUP/sMUPs were around 40 % attenuated and the deeper MUP/sMUPs were 48 % attenuated. Similar data from older men suggest around 20 % greater attenuation of the signal compared with young.

**Fig. 3:**
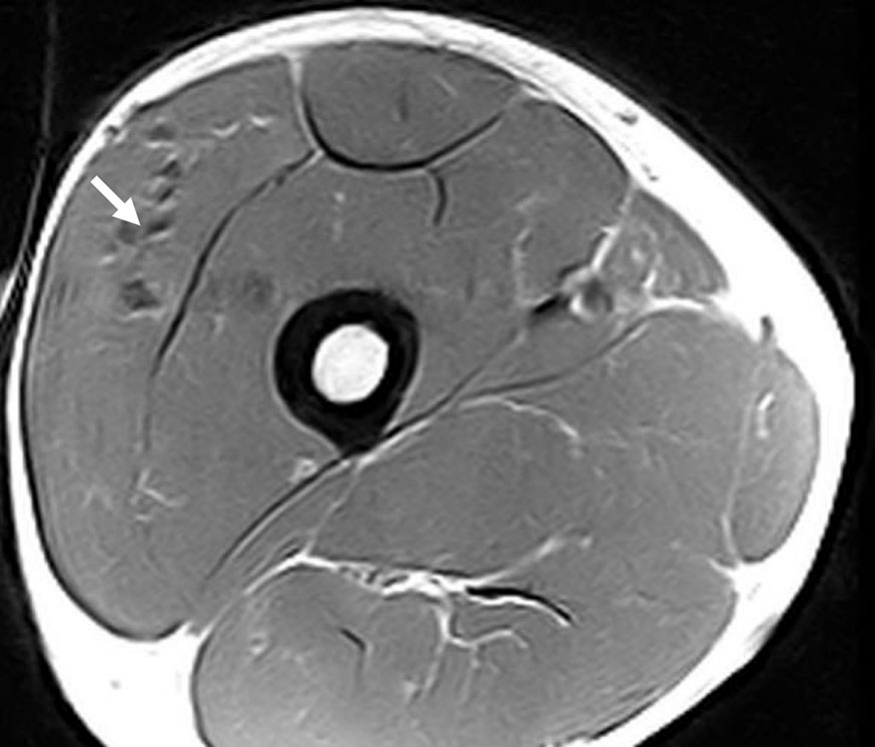
MRI image from a young man showing connective tissue accumulations around the vastus lateralis motor point (*arrow*). The surface-recorded CMAP was 66 % smaller, the sMUP was 60 % smaller and the MUPs were 46 % larger than average for young men

The greater attenuation in old is likely related to connective tissue or adipose tissue deposits in the muscle. An example is shown in Fig. 3a to illustrate the effects of muscle composition on EMG signals. The MRI and corresponding MU data were collected around 6 years after two muscle biopsy samples were taken from close to the proximal motor point. The participant had values within the normal range for the CMAP and sMUP (20 and 35 % lower than average young) and for the MUPs (10 % higher than average young) at the distal VL motor point where tissue damage was not present. At the proximal motor point around the site of damage, the CMAP and sMUP were 66 and 60 % lower, while the MUPs were 46 % larger than average for young men. The subject had MUNE values that were 1 and 7 % higher than average young men at the proximal (damaged) and distal VL motor points.

The extent of attenuation may differ between old and young or, indeed, between individuals due to changes in muscle and subcutaneous tissue composition, but the attenuation affects both CMAP and sMUP similarly and consequently MUNE values are probably not compromised during normal ageing.

### sEMG recording area and muscle size

The reported CMAP values for a variety of small muscles are surprisingly similar to those reported for larger muscles (Galea 1996; McNeil et al. 2005; Dalton et al. 2008; Power et al. 2010, 2012). In large muscles the CMAP is smaller than would be expected if it were the summation of the electrical activity of all MUs within the muscle. This suggests that the CMAP, and by extension the MUNE values, of the larger muscles is not an estimate of the number of MUs within the whole muscle but is, instead, a representation of MUs in the volume of muscle ‘seen’ by the surface electrode. This is also apparent by comparing MUNE values reported for individual muscles against anatomical counts of spinal motor neurons. MUNE values derived from leg muscles (albeit, mainly smaller muscles) are often around 200–400 and even these are likely to be on the high side as a result of preferential sampling of smaller units during STA-MUNE. However, they are clearly some way short when considered against the anatomical counts by Tomlinson and Irving (1977) who estimated around 60,000 motor neuron cell bodies in the lumbo-sacral region (innervating the legs) of the spinal cord of young men and 40,000 in older men.

It is not easy to know precisely what volume of muscle is captured by the surface electrode, but one study estimated it to be from a depth of around 2 cm (Barkhaus and Nandedkar 1994). If this is a radius of a hemisphere of muscle (~16 cm^3^), it is a very small proportion (0.008) of the total volume of large muscles such as the quadriceps (~2000 cm^3^). If a nominal MUNE of 300 is adjusted for this proportion, the total number of MUs is around 37,500. This value is high, most likely due to preferential sampling of smaller MUs during STA-MUNE.

## Age-related motor unit remodelling assessed by indwelling electrodes

Using the incremental stimulation MUNE technique, Campbell et al. (1973) noted that older subjects tended to have larger sMUPs compared with the young. Other studies using iEMG and voluntary contractions similarly reported larger MUPs in old during low and moderate force contractions (Fig. 2).

It is important to record MUPs from several locations in the muscle in order to sample representative areas (Brown et al. 1988; Ives and Doherty 2014). Recording MU potential trains reveals the extent of MU remodelling and the stability of neuromuscular junction transmissions, evidenced in terms of MUP area or amplitudes, distribution of MUP sizes, number of turns, number of phases and jiggle (Stashuk 1999a, b; Abdelmaseeh et al. 2014).

Figure 2 shows MUPs reported in various healthy, older muscles when expressed as a percentage of MUPs from young. Extending the observation of larger sMUPs in the EDB of older people made by Campbell et al. (1973), the incremental stimulation technique was later used to show larger sMUPs in the thenar and EDB, but sMUPs in the BB were around 25 % smaller in old compared with young (Galea 1996). A macro EMG technique was developed to more accurately reflect the MU size from iEMG-recorded MUPs of all muscle fibres within a MU. Similar to STA, signals from the cannula of the needle are triggered by intramuscular recordings, thereby increasing the recording area making it more likely to capture an entire MUP (Stålberg 2011). This technique showed almost two-fold larger MUPs in older TA and VL compared with young, while the BB was increased by around 30–50 % (Stalberg and Fawcett 1982). Data from concentric needles show larger MUPs in older soleus across a range of voluntary contraction intensities (Dalton et al. 2008), and larger MUPs have also been reported in TA, vastus medialis (Hourigan et al. 2015) and VL (Piasecki et al. 2015) with this technique.

Hourigan et al. (2015) reported ‘near-fibre’ (NF) data in healthy older subjects, which records from small numbers of single fibres located close to the needle in order to more accurately detect MU potentials. They showed higher NF jiggle in the TA and vastus medialis of nine older compared with nine younger men. MUNE values were lower in the TA, but were not estimated for the vastus medialis (Hourigan et al. 2015). A similar technique applied to the VL of 22 young and 20 old similarly showed 11 % higher NF jiggle in old and 30 % lower MUNE values compared with young (Piasecki et al. 2015), which is in line with previous studies of VL that showed greater complexity of MUs using single-fibre EMG and Macro EMG (Stalberg 1979; Stalberg and Fawcett 1982). Higher jiggle is thought to occur due to increased transmission variability from unstable neuromuscular junctions within individual MUs (Stålberg and Sonoo 1994). These MU changes indicate discharge variability and asynchronous action potential transmission within the same MU (Nandedkar et al. 1988), possibly reflecting alterations in neuromuscular junction structure or function or variability in conduction along peripheral axonal branches.

Given the uncertainty about the MUNE values that rely on sEMG measures (discussed above), an alternative would be to use MUPs alongside measures of muscle size to make an estimation of *proportional* changes to MU numbers. This can be achieved by simply dividing muscle anatomical CSA (e.g. measured by magnetic resonance) by the average MUP (measured using intramuscular EMG). Published data from Macro EMG give the average VL MUP as 970 and 760 (µV ms) in old and young, respectively (Stalberg and Fawcett 1982). The average anatomical CSA of the VL (at mid-muscle belly) is 23 and 30 cm^2^ in old and young men, respectively (Maden-Wilkinson et al. 2013). From these retrospectively fitted data it is estimated that older subjects have 60 % of the MUs of the young, and using prospective data, Piasecki et al. (2015) estimated that otherwise healthy older men had around 50 % of the MUs of young in VL. These 40–50 % MU losses are slightly greater than the averages presented in Fig. 2, which are derived from MUNE and therefore relied upon surface-recorded sMUPS and CMAP (for which attenuation affects the signals and the values are not corrected for muscle size), and greater than the ~30 % motor neuron loss estimated from small numbers of autopsy specimens (Tomlinson and Irving 1977).

### Which MUs are preferentially lost during healthy ageing?

The reduction in MU numbers, together with an increase in average MU size, could come about either as a result of the smaller units being lost or, as is generally believed, a loss of motor neurons supplying the larger MUs combined with reinnervation of the denervated fibres by sprouting of axons from surviving motor neurons. There is little indication either way from the EMG data. A modest reduction in MU discharge rates was reported in healthy older subjects in the TA (Connelly et al. 1999; Patten et al. 2001; Klass et al. 2008), FDI (Kamen et al. 1995), VL (Piasecki et al. 2015) and soleus (Dalton et al. 2008), but others reported no difference between young and old in TA, BB or VL in MU discharge rates (Roos et al. 1999; Power et al. 2010, 2012). Slower discharge rates may be due to the recruitment of fewer, but larger slow-phenotype MUs during moderate intensity contractions. If the low threshold MUs did indeed have increased propensity for reinnervation of orphaned fibres, then their MUPs should be proportionately larger than the later-recruited higher threshold MUs. One of the few studies to report MUPs, sMUPs and MUNE at a range of contraction intensities was by Dalton et al. (2008). They showed that older muscles had around 60 % larger MUPs during all measurements collected from a range of intensities including very low through to 30 % MVC in soleus. This indicates that older people have larger MUs compared with young across a broad range of recruitment thresholds. However, in this study (Dalton et al. 2008) the sMUPs tended to be smaller in old compared with young at higher intensity contractions, which is not consistent with the MUP data and may be due to increased signal attenuation in the old, as discussed above.

There is a skewed distribution of MU types in muscles like VL or FDI that have around 50 % type I and 50 % type II in the overall muscle cross section. Enoka and Fuglevand (2001) explained that 84 % of MUs were type I, despite the type I fibre area being just 50 % of the overall FDI muscle area. Thus, loss of just one of the fast MUs with high innervation ratio would have little impact on MU numbers but potentially impacts greatly on fibre losses. Lexell et al. (1988) suggest similar numbers of type I and type II fibres are lost with ageing, so assuming random reinnervation of the different fibre types, this would indicate substantially greater losses of smaller MUs compared with large. The MU lost, large or small, might affect the success of reinnervation, with the fewer fibres of smaller MUs possibly more easily being accommodated into other units, but very little experimental evidence exists.

## Motor unit remodelling and control of movements

### Muscle size and strength

**Fig. 4:**
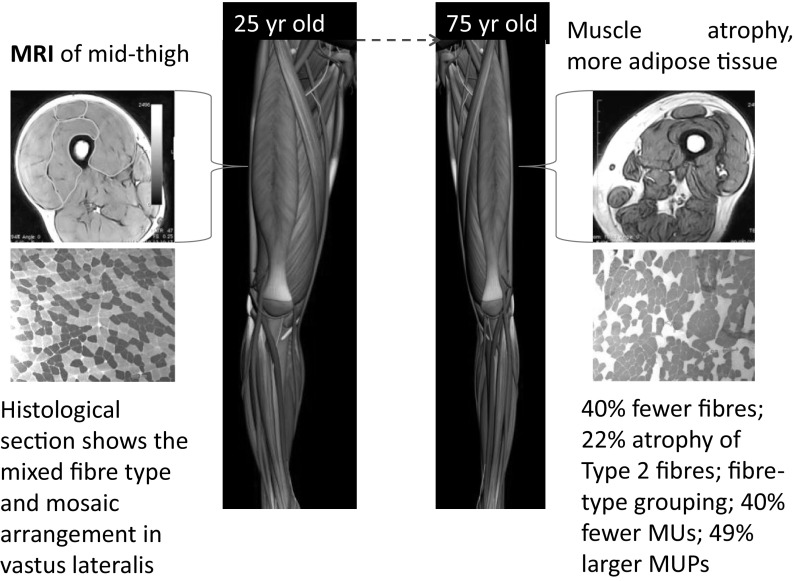
Atrophic muscles in older age. Compared with young, a typical healthy 75 year old man has around 15 % lower appendicular lean mass (McPhee et al. 2013); 30 % smaller knee extensor muscles (Maden-Wilkinson et al. 2014); 35 % lower knee extension strength (McPhee et al. 2013) and 35 % lower leg power (Stenroth et al. 2015); 20–40 % fewer muscle fibres in the VL, fibre-type grouping and small, angular fibres (Lexell et al. 1988; Lexell and Downham 1991)

Figure 4 provides a summary of muscular changes during healthy ageing. Dual-energy X-ray absorptiometry is most commonly used to assess muscle (or ‘lean’) mass, but it underestimates the extent of muscle loss during ageing (Maden-Wilkinson et al. 2013, 2014). MU remodelling contributes to the muscle losses, but it remains unclear whether any particular MUs, small or large, are preferentially lost or which are enlarged during normal ageing. Motor neuron loss leaves the muscle fibres within the MU denervated, but some are ‘rescued’ by sprouting of nearby neuron branches. The reinnervation process gives enlarged MUPs, increased fibre density (Stalberg and Thiele 1975; Stalberg 1982; McComas et al. 1993; Luff 1998) and fibre-type grouping (Lexell and Downham 1991) with healthy older age.

The rescue of denervated fibres helps to preserve total muscle mass and maximal force generating capacity in the face of extensive motor neuron losses. Therefore, MU loss must precede clinically-relevant muscle losses such as sarcopenia or dynapenia (Piasecki et al. 2015), but longitudinal data are not available to confirm this. The re-innervation process is, however, limited, as post-mortem studies showed that around 30–40 % of fibres in VL are lost by age around 75 years (Lexell et al. 1988; Lexell and Downham 1991), with both type I and type II fibre losses being the major cause of muscle atrophy with healthy ageing, rather than atrophy of individual fibres (Lexell et al. 1988).

MU loss was stated as a cause of muscle weakness in older BB using MUNE (Doherty et al. 1993). A study using MUNIX showed loss of MUs in abductor pollicis of people aged around 67 years was associated with lower pinch strength (Kaya et al. 2013). Drey et al. (2013) applied the MUNIX method to the thenar muscle of 27 sarcopenic patients and found the MUNIX values to be lower than those in non-sarcopenic old. They also reported that 25 % of the sarcopenic patients had ‘pathological’ MUNIX and motor unit size index (MUSIX) values. While these studies indicate functional and structural consequences of MU remodelling, there are issues when defining sarcopenia (Reijnierse et al. 2015) and possible problems of attenuation and cross-contamination when using MUNIX alone.

### Control of movements

Coordinated movements require not only the proper involvement of different muscles, but also the correct recruitment and activation of MUs in the individual muscles. MU size may be described relative to the innervation ratio or the axonal diameter, and in young healthy muscles the two are likely to correlate well, however older muscle tends to have an increased innervation ratio due to remodelling but without necessarily the increased axonal diameter. This results in not only larger MUs with a low recruitment threshold, but also grouping of muscle fibres, slowing of firing rates and instability of neuromuscular junction transmissions. This neuromuscular remodelling is expected to have implications for fine motor control. It is notable in this respect that increased tremor, slower walking speeds and poor balance are common complaints of the elderly. It is not a straightforward task to link poor mobility and balance directly to MU remodelling, since so many other factors contribute to mobility including eyesight, vestibular function, proprioception and motor output (Luu et al. 2012), which also deteriorate with ageing.

The loss of MUs has not been systematically examined in association with function in large muscles, but there are some indications of control deficits from studies of small hand muscles where MU remodelling was associated with reduced coordination of finger movements (Galganski et al. 1993; Erim et al. 1999; Burnett et al. 2000; Semmler et al. 2000). Thus, more work is needed to establish the functional consequences of extensive MU remodelling in older age, particularly in the large leg muscles and investigations using longitudinal studies.

## Exercise as a possible intervention strategy

Since motor neurons are terminally differentiated, the large number that are lost during healthy ageing can never be recovered. It is, therefore, important from the public health perspective to find ways to prevent the losses from occurring, or to find ways to help older people cope with any deficits in MU numbers and function.

Skeletal muscle mass and strength are responsive to exercise even in older age, increasing after regular high force contractions, but decreasing after disuse. Maintaining exercise through middle and older age helps to preserve neuromuscular function, particularly at the neuromuscular junction (reviewed by (Deschenes 2011; Nishimune et al. 2014). A study using STA-MUNE showed that 10 older endurance runners aged around 64 years had similar MU numbers to young adults in their TA, while non-athletic older adults aged around 66 years had fewer MUs than the young; however, when the BB was examined, the masters athletes had similar low MU numbers as the old (Power et al. 2010, 2012). This was taken as evidence of exercise-related preservation of the specific MUs within the muscles most often used during exercise. This notion of “use it or lose it” is very appealing, but the small sample size and cross-sectional study design mean that additional studies are needed.

In spite of extensive MU losses and remodelling, exercise training is known to improve movement control and balance in older people, resulting in fewer falls (Sherrington et al. 2011). Various exercise routines have proven effective for balance and falls prevention (Rubenstein et al. 2000; Orr et al. 2006; Gillespie et al. 2012; Franco et al. 2014), but the greatest benefits come from specific balance training (Sherrington et al. 2011). Improved modulation of rate coding was observed with practice of performing a precision task with a hand muscle (Knight and Kamen 2004). The extent to which precision and balance adaptations are due to improved MU recruitment and activation strategies remains unknown for larger muscles. Such improvements to MU recruitment and activation may help older people to cope in the face of significant motor neuron losses.

## Conclusion

Healthy older people have around 40 % lower MUNE values compared with young. The surviving low- and moderate-threshold MUs are around 50 % enlarged and show increased complexity and fibre density as well as instability of the neuromuscular junction transmissions. There is considerable inter-individual variability in MU characteristics at all ages and this may lead to sampling bias in studies that included small numbers of participants. The neural changes may precede clinically-relevant muscle wasting, weakness and reduced coordination of movements. It remains unclear whether lifelong exercise can help to preserve MU numbers or how exercise training modulates MU function to help older people cope with existing MU deficits.
